# Comparison of various aspects of women’s lives between infertile and women with and without tubal ligation: a comparative cross-sectional study

**DOI:** 10.1186/s12905-021-01454-7

**Published:** 2021-08-28

**Authors:** Mahnaz Ashrafi, Shahideh Jahanian Sadatmahalleh, Negin Mirzaei, Nadia Jahangiri, Samaneh Youseflu, Malihe Nasiri

**Affiliations:** 1 Department of Endocrinology and Female Infertility, Reproductive Biomedicine Research Center, Royan Institute for Reproductive Biomedicine, ACECR, Tehran, Iran; 2 Department of Obstetrics and Gynecology, Faculty of Medicine, Iran University of Medical Science, Tehran, Iran; 3 Department of Reproductive Health and Midwifery, Faculty of Medical Sciences, Tarbiat Modares University, Tehran, Iran; 4 Department of Midwifery, School of Nursing and Midwifery, Zanjan University of Medical Science, Zanjan, Iran; 5 Department of Basic Sciences, Faculty of Nursing and Midwifery, Shahid Beheshti University of Medical Sciences, Tehran, Iran

**Keywords:** Infertility, Tubal ligation, Sexual function, Quality of life, Depression

## Abstract

**Background:**

The aim of this study is to compare anxiety, depression, body image, self-esteem, sexual function, and quality of life (QoL) between infertile women and control fertile women undergoing tubal ligation (TL) and using condom.

**Methods:**

This cross-sectional study was conducted on 600 women in three groups of infertile and control fertile women with or without TL (200 women in each group), who met the inclusion criteria. They were selected from Royan Institute and a number of health care centers in Tehran (Iran) from May 2017 to February 2019. The subjects were asked to fill out the Short Form Health Survey (SF-12), Female Sexual Function Index (FSFI), Hospital Anxiety and Depression Scale (HADS), Body Image Concern Inventory (BICI), and Rosenberg’ Self-Esteem Scale (RSES). One-way ANOVA was used to identify the possible statistical differences between the three groups of participants.

**Results:**

The mean scores of all FSFI domains were lower in the control TL women, and the differences between the three groups in all dimensions were statistically significant. In addition, the TL group had more female sexual dysfunction (FSD) comparing to the infertile and condom group (22.43 ± 5.30, 24.79 ± 4.74, and 28.03 ± 3.29, respectively *P* <  0.001). There was a significant difference between the three groups in SF-12 scores (76.59 ± 13.14, 68.49 ± 14.47, and 78.87 ± 12.62, respectively *P* < 0.001). Also there was a significant difference between the three groups in anxiety, depression, and total scores of HADS (*P*  < 0.001). Furthermore, infertile women had lower body image (*P*  < 0.05) and the TL group had lower self-esteem comparing to the two other groups (*P*  < 0.05).

**Conclusions:**

The findings revealed the adverse effects of using TL on the anxiety, depression, sexual life, body image, and QoL of women. It is recommended that health-care professionals should increase their awareness and knowledge regarding the side-effects of using TL on women’s lives and share this information with the patients.

## Background

Childbearing is one of the main goals of family formation in almost all nations worldwide. However, it has a special socio-cultural and religious significance in Iranian culture. As a matter of fact, it is an important expected outcome of sexual relationship for most couples. Many factors that may disrupt the natural fertility process can impose an enormous psychological burden on a couple’s life [1, 2].

Infertility is the inability to conceive after 12 months of frequent coitus without using contraceptives. About 25% of couples in the developing countries suffer from this problem [3]. Infertility has negative effects on various aspects of couples’ lives [4] and can increase marital conflicts and Sexual Dysfunction (SD), especially in females [5]. Anecdotal evidence suggests that infertility as a stressful condition doubles the risk of anxiety and depression in women [6].

Tubal ligation (TL), which is the most commonly used family planning method worldwide, is a surgical procedure for contraception in women who have completed their family or have no desire to become pregnant [7]. Despite using TL by 19% of women aged between 15 and 49 years [8], sexual functioning and other aspects of QoL in women undergoing TL have not been adequately explored and available studies have found conflicting results. Some researchers have reported destructive effects associated with TL on QoL and sexual function [9–13].

Childbearing can be affected by both infertility and TL. Although, most of infertile couples naturally have desire to have children of their own but they are unable to do so. Therefore, they usually seek some type of medical infertility treatment. On the other hand, TL is a voluntary choice for those who no longer want children and tend to limit their family size [14].

This study aimed to compare depression, anxiety, body image, self-esteem, sexual function, and QoL in infertile and fertile women (condom and TL).

## Methods

### Participants

The present comparative cross-sectional study was conducted between May 2017 and February 2019. The sample consists of infertile women referring to Royan Research Institute in Tehran (Iran) as a referral center, and two control groups of reproductive age women using TL or condom referring to four randomly selected health care centers of different geographic regions (East, West, North and South) in Tehran City (Iran) for unrelated problems. The sampling was done using the convenience sampling method.

The sample size was estimated 654 (218 women in each group) using the following formula (confidence coefficient of 99% and power of 90%) by considering 25% sample loss:

$$\overline{\mu } = {1}/{\text{k}}\sum \mu_{{\text{j}}}$$

$$\Delta = {1}/\sigma^{{2}} \sum \left( {\mu_{{\text{j}}} - \overline{\mu }} \right)^{{2}}$$

$${\text{N}} = \lambda /\Delta = {17}.{43}/0.{1} = {174}.{3 - 175}$$

where k is the number of study groups (N = 3), J = 1,2,3, µ1,µ2,µ3 indicates the mean score in the study groups, $\overline{\mu }$ and σ represent, the overall mean and standard deviation, respectively, and λ is equal to 17.43 [15]. Sample size calculation was done using the PASS software.

In this study, the number of participants in each group were 200 women (total = 600) who met the inclusion criteria. Overall, the inclusion criteria included women being at the reproductive age (≤ 35 years), married, living with their husbands, and sexually active (had sexual intercourse in the past four weeks). Women who were unable to conceive after 12 months of frequent coitus without using contraceptives and had not entered a treatment cycle yet were listed as the infertile group. Additionally, women who had undergone TL for longer than 1 year and women who had used condoms to prevent pregnancy were included as the two control study groups. To avoid possible confounding factors, the exclusion criteria included infertile couples with male infertility, having gynecological disorders, suffering from chronic diseases (such as diabetes, hypertension, etc.), not being sexually active, breastfeeding, and taking any antidepressant drugs, serotonin and norepinephrine reuptake inhibitors (SNRIs), or antipsychotic drugs that could have sexual side effects.

The research was conducted using a standard questionnaire namely, Hospital Anxiety and Depression Scale (HADS), Female Sexual Function Index (FSFI), Body Image Concern Inventory (BICI), Rosenberg’ Self-Esteem Scale (RSES), and Short Form Health Survey (SF-12) were used to measure the various aspects of the participants’ lives. Before being asked to fill out the questionnaires, the respondents were provided with explanations and the approval letter. The study protocol was approved by the Institutional Review Board and the Ethics Committee of Royan Institute (IR.ACECR.ROYAN.REC.1395.97).

### Questionnaires

The participants were asked to complete several self-report questionnaires as follows:

demographic survey that included the participants’ age number of children, educational level, occupational status, Body Mass Index (BMI), smoking status, type of previous deliveries, menstruation status, and husband’s age.

#### Anxiety and depression

The HADS objectively measures anxiety and depression. It contains a 7-item depression scale and a 7-item anxiety subscale (the score range of each component is 0–3). Higher scores represent higher symptom levels for both subscales and a score of 11 and above is considered a clinical disease [16]. The validity and reliability of the Persian version of HADS has been confirmed [17].

#### Female sexual function

FSFI investigates the quality of a woman’s sexual life over the past four weeks prior to a study. This questionnaire measures desire, arousal, lubrication, satisfaction, orgasm, and pain by asking 19 questions that are rated on a 5-point scale. The domain score of 0 indicates that the respondent had no sexual activity during in the last month. A total score of less than 23 denotes sexual dysfunction, and scores less than 3.3 in the desire domain, less than 3.4 in the arousal and orgasm domains, less than 3.7 in the lubrication domain, less than 3.8 in the satisfaction and pain domain are used to classify the participants as having difficulties in the corresponding domains [18]. The Persian version of FSFI has been evaluated for both reliability and validity [19].

#### Body image

Development of the Body Image Concern Inventory (BICI) is used to assess the participants’ dysmorphic concerns. It consists of 19 questions that examine the individuals’ dissatisfaction and concerns about their appearance. The answers are based on the Likert scale of 1–5 and the total score range is 19–95; higher scores indicate higher levels of dissatisfaction about one’s body image or appearance [20]. The validity and reliability of this questionnaire have been previously assessed in Iran [21].

#### Self-esteem

Rosenberg’ Self-Esteem Scale (RSES) is composed of 10 questions that are scored on a 4-point Likert-type scale, ranging from 1 (totally disagree) to 4 (totally agree). A higher score indicates higher self-esteem [22]. Validity and reliability tests of the Iranian form of this scale have been previously performed [23].

#### Quality of life

The participants were asked to fill out the Short Form Health Survey (SF-12) that measures QoL in eight areas: physical functioning, physical role, bodily pain, general health, vitality, social functioning, role emotional, and mental health by 12 items. Higher scores indicate better QoL [24]. The validity and reliability of this questionnaire have been confirmed in Iran [25].

### Statistics

All statistical analyses were performed by the SPSS software (ver. 25.0) (SPSS Inc., Chicago, IL, USA). A one-sample Kolmogorov–Smirnov’s test was used to analyze normality for the continuous variables, which were compared using the Student’s t-test and x^2^-test.

One-way ANOVA and LSD post-hoc test were employed to compare each variable between the three study groups. Differences were considered significant at *P*  <  0.05 for the two tails.

## Results

**Fig. 1:**
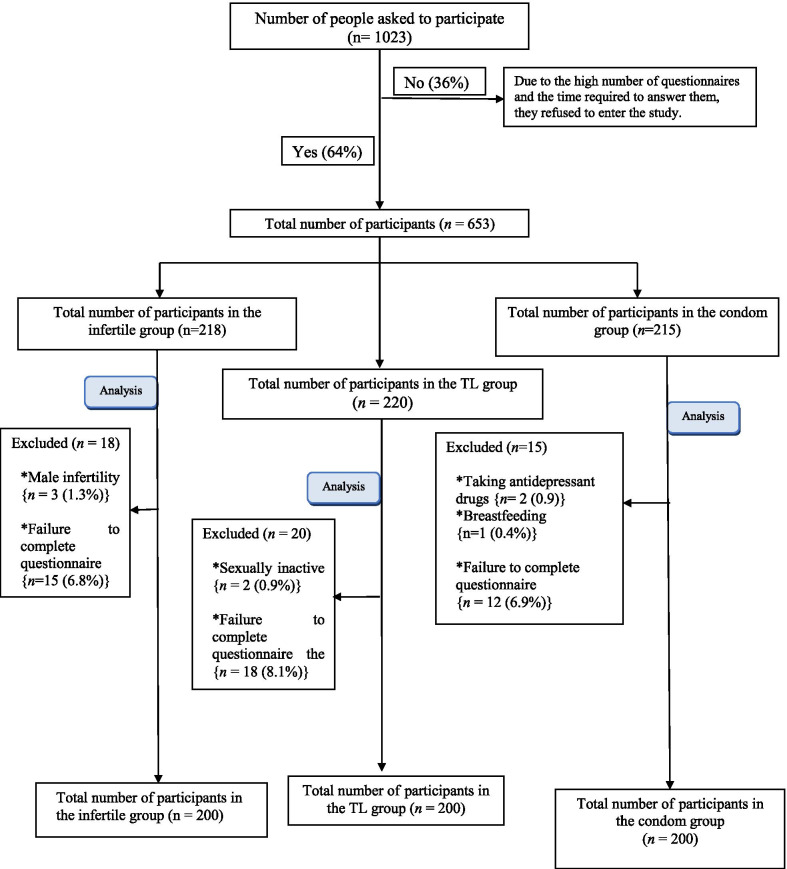
Flow chart of the study

Overall, 370 out of the 1023 potentially eligible women, who were asked to complete the questionnaires, refused to participate in the study due to the long time needed for completion of the questionnaires, so the response rate was approximately 64%. A total of 653 women were enrolled into the study, 53 were excluded because of male infertility (n = 3), sexual inactivity (n = 2), taking antidepressant (n = 2), breastfeeding (n = 1), and incomplete questionnaires (n = 45). Additionally, the rate of sample loss was 8% (Fig. 1). Finally, 200 women were enrolled in each group.

Table 1 describes the characteristics of women in the infertile, TL, and condom groups. The age of the participants ranged from 20 to 43 years. As shown in Table 1, there are no significant differences (*P* > 0.05) between the women’s age groups, BMI, smoking status, educational level, type of previous deliveries, and age of husband. However, there are statistically significant differences among the groups in terms of menstrual cycle length (28.71 ± 2.08, 27.99 ± 5.95, and 29.43 ± 6.49, P = 0.02) and specifications (*P* < 0.001). The number of children in the infertile, TL and condom group were 0.83 ± 0.38, 2.35 ± 0.56, and 2.29 ± 0.71, respectively, showing a statistically significant difference between them (*P* < 0.001).

**Table 1:** Comparison of demographic characteristics between infertile, TL and condom groups

Variable	Infertile group n = 200	TL group n = 200	Condom group n = 200	*P* value
Age of female*	32.48 ± 4.92	33.06 ± 3.01	33.07 ± 3.61	0.25
Number of children*	0.83 ± 0.38	2.35 ± 0.56	2.29 ± 0.71	< 0.001
Education of female**				0.08
Illiterate	0 (0)	1 (0.5)	1 (0.5)	
Under diploma	85 (42.3)	111 (55.5)	99 (49.5)	
Higher diploma	116 (57.7)	88 (44)	100 (50)	
Age of husband*	39.06 ± 6.27	38.93 ± 8.19	38.22 ± 4.27	0.17
Job of female**				0.075
Housewife	148 (73.6)	166 (83)	157 (78.5)	
Recruitment	53 (26.4)	43 (17)	43 (21.5)	
BMI (kg/ m^2^)*	27.02 ± 4.39	28.05 ± 4.06	27.43 ± 4.81	0.06
Smoking**				0.60
Yes	1 (0.5)	1 (0.5)	0 (0)	
No	200 (99.5)	199 (99.5)	200 (100)	
Menstrual cycle feature**				< 0.001
Regular	154 (77)	168 (84)	187 (93.5)	
Irregular	46 (23)	32 (16)	13 (6.5)	
Menstrual cycle length (days)*	28.71 ± 2.08	27.99 ± 5.95	29.43 ± 6.49	0.02
Type of delivery**				0.15
Cesarean section	19 (79.2)	152 (76)	136 (68)	
NVD	5 (20.8)	48 (24)	64 (32)	

BMI: Body Mass Index, NVD: Natural Vaginal Delivery

*Values are given as mean ± SD using t-test

** Values are given as a number (%) using Chi-squared test

### Sexual function status

The evaluation of FSFI scores and comparison of sexual dysfunction are shown in Table 2. As can be seen, all mean values are lower in the TL group (except orgasm). Also the differences in scores among the three groups are statistically significant in all domains. There is a statistically significant difference among the infertile, TL and condom groups in terms of desire, arousal, lubrication, orgasm, satisfaction, pain, and total FSFI scores. In addition, the prevalence of sexual dysfunction in the women with TL is significantly higher than in the infertile and condom groups (*P* < 0.001).

**Table 2:** Comparison of FSFI scores and dysfunction between infertile, TL and condom groups

Variable	Infertile group (I) N = 200 Mean ± SD	TL group (T) N = 200 Mean ± SD	Condom group (C) N = 200 Mean ± SD	*P* value*	Pair-wise comparison *P* value**
Desire	3.79 ± 0.83	3.07 ± 0.84	3.99 ± 0.75	< 0.001	I and C: 0.012 I and T: < 0.001 T and C: < 0.001
Arousal	3.99 ± 1.08	3.28 ± 1.01	4.20 ± 0.82	< 0.001	I and C: 0.003 I and T: < 0.001 T and C: < 0.001
Lubrication	4.61 ± 1.20	3.82 ± 1.19	4.91 ± 0.76	< 0.001	I and C: 0.005 I and T: < 0.001 T and C: < 0.001
Orgasm	3.06 ± 0.91	3.81 ± 1.30	4.89 ± 0.77	< 0.001	I and C: < 0.001 I and T: < 0.001 T and C: < 0.001
Satisfaction	4.74 ± 1.12	4.19 ± 1.15	5.14 ± 0.88	< 0.001	I and C: < 0.001 I and T: < 0.001 T and C: < 0.001
Pain	4.67 ± 1.28	4.18 ± 1.44	4.87 ± 0.96	< 0.001	I and C: 0.102 I and T: < 0.001 T and C: < 0.001
Total score	24.79 ± 4.74	22.43 ± 5.30	28.03 ± 3.29	< 0.001	I and C: < 0.001 I and T: < 0.001 T and C: < 0.001
Dysfunction***				< 0.001	
Yes	148 (73.6)	177 (88.5)	115 (57.5)		
No	53 (26.4)	23 (11.5)	85 (42.5)		

FSFI: Female Sexual Function Index, TL: tubal ligation. ANOVA: analysis of variance

*One-way ANOVA

**One-way ANOVA followed by LSD post-hoc test

***Values are given as a number (%) using Chi-squared test

### Quality of life status

Table 3 compares the SF-12 scores between the infertile, TL and condom groups. The mean of total SF-12 scores was significantly lower in the TL group compared with the other two groups (76.59 ± 13.14, 68.49 ± 14.47, and 78.87 ± 12.62, respectively, *P*  < 0.001).

**Table 3:** Sum and total scores for the domain subgroups of QoL, HADS, and body image between infertile, TL, and condom groups

Variable	Infertile group (I) n = 200 Mean ± SD	TL group (T) n = 200 Mean ± SD	Condom group (C) n = 200 Mean ± SD	*P* value*	Pair-wise comparison *P* value**
*SF-12*					
Sum score of physical components (PCS-12)	78.96 ± 14.10	67.88 ± 15.87	79.52 ± 14.25	< 0.001	I and C: 0.70 I and T: < 0.001 T and C: < 0.001
Sum score of mental components (MCS-12)	74.22 ± 15.23	69.10 ± 16.02	78.23 ± 13.34	< 0.001	I and C: 0.007 I and T: < 0.001 T and C: < 0.001
Total score	76.59 ± 13.14	68.49 ± 14.47	78.87 ± 12.62	< 0.001	I and C: 0.09 I and T: < 0.001 T and C: < 0.001
*HADS*					
Depression	5.29 ± 3.22	6.65 ± 3.57	4.42 ± 3.22	< 0.001	I and C: 0.09 I and T: < 0.001 T and C: < 0.001
Anxiety	7.66 ± 3.05	10.05 ± 3.93	6.84 ± 3.27	< 0.001	I and C: 0.005 I and T: < 0.001 T and C: < 0.001
Total score	12.88 ± 4.93	16.55 ± 6.49	11.23 ± 5.97	< 0.001	I and C: 0.017 I and T: < 0.001 T and C: < 0.001
Body image	36.58 ± 12.4	33.86 ± 13.1	32.43 ± 10.35	0.02	I and C: 0.001 I and T: 0.024 T and C: 0.234
Self-esteem	29.16 ± 5.26	25.01 ± 5.44	29.16 ± 5.52	< 0.001	I and C: 0.881 I and T: < 0.001 T and C: < 0.001

QoL: Quality of Life, SF-12: Short Form-12, HADS: Hospital Anxiety and Depression Scale, TL: Tubal Ligation

*One-way ANOVA

**One-way ANOVA followed by LSD post-hoc test

The information summarized in Table 3 reveals that the TL group has significantly less physical, psychological, and total scores of QoL than the other two groups (*P*  < 0.001). There is also no significant difference between the infertile and condom groups in the physical and total scores of QoL (*P* > 0.05).

### Anxiety and depression

As shown in Table 3, there are significant differences between the mean scores of depression, anxiety, and total HADS in the infertile, TL and condom groups (12.88 ± 4.93, 16.55 ± 6.49, and 11.23 ± 5.97, respectively, P < 0.001). Significant differences were observed between the TL group and the infertile and condom groups in depression, anxiety, and total scores of HADS. However, there was no significant difference between the infertile and condom groups in terms of depression (*P* > 0.05).

### Body image and self-esteem

It can be seen from Table 3 that the body image score difference is significant among the TL, infertile and condom groups (*P*  < 0.05) and the infertile women have a higher score (36.58 ± 12.40, 33.86 ± 13.10, and 32.43 ± 10.35, *P* = 0.02). Self-esteem was statistically lower in the infertile women comparing to the other two groups (29.16 ± 5.26, 25.01 ± 5.44, and 29.16 ± 5.52, respectively, *P* < 0.001).

## Discussion

Due to the increasing rate of infertility in Iran, the main goal of the current study was to assess SF, QoL, depression, anxiety, self-esteem, and body image among infertile women and compare the results with two control groups (TL and condom). Also, because of the soaring use of TL by Iranian women in recent years, it is important to evaluate potential complications associated with this method. Diagnosis of sexual dysfunction and impairment of QoL, indicate a need for sufficient education and comprehensive consultation by the healthcare system prior to implementing the procedure.

### Anxiety and depression

Although TL is the most commonly used form of contraceptive method worldwide, its long-term psychological effects are still obscure. Contrary to expectations, the results of this study showed that anxiety and depression symptoms in the women undergoing TL surgery would be higher than that in infertile women.

There is a huge amount of evidence indicating that depression and anxiety are more prevalent among infertile women comparing to non-infertile women. Almost half of the infertile subjects have moderate to severe symptoms of depression and the level of anxiety increases by infertility [26]. Anxiety and depression caused by infertility can be due to various factors such as uncertainty about the cause of infertility, duration of treatment, and unspecified treatment, as well as financial and social pressures. Since cyclical hormonal changes are related to anxiety and depression, these observations may be due to undergoing TL [27]. Lin et al. [28] suggested sterilization as a risk factor for depression and anxiety. Also the risk of depression and anxiety following TL was reported to be 2.34 and 2.88 times greater than that before TL, respectively. A similar study conducted on 169 women who had experienced TL reported increased Beck depression inventory scores after TL. In addition, the participants were suffering from regret after sterilization [27]. Complications by reason of TL cause regret [14]. In traditional societies like Iran, the picture of a woman is directly related to her fertility and motherhood ability; this causes her to feel perfect and valuable. Although TL is a voluntary procedure, cultural factors that are rooted in one's unconscious cause convey her feelings of inadequacy and unattractive that can manifest as sexual dysfunction and regret [9]. It has been shown in several studies that anxiety and depression are more common in women regretting sterilization in comparison to the control group [14, 27].

Depression can be influenced by many factors, including a feeling of guilt after an irreversible surgery, negative perceptions of others, a change in husband's intuition, cultural, and religious differences, or previous emotional disorders. Women should be given time to consider their decision on fertility/infertility and be provided with psychological support during this time.

### Body image and self-esteem

The infertile women had lower body image in comparison with the TL and condom groups. This finding confirms the association between body image and infertility. In addition to physical appearance, a person's body image also reflects his/her physical well-being and biological status [29]. Previous studies have shown that self-concept, identity development, anxiety, and depression are factors closely related to a woman’s body image that might be affected by infertility [30]. A strong relationship between infertility and depression has been reported in the literature [29]. Depression in different stages of life may lead to a distorted body image [30]. In the present study, self-esteem scores were lower in the TL women as compared to the other two groups. However, many authors have reported no link between TL and self-esteem [11, 31].

This observation may support the hypothesis that although individuals continuously think about their appearances, after a physical disease like infertility, they become more aware and alert of their bodies and are more mentally concerned [32].

In sum, very little research has been done on the relationship between undergoing TL and body image. Li et al. [11] and Raine et al. [31] found that TL had no effect on body image. Further work is required to establish the validity of this result and different factors affecting the body image.

### Sexual function status

The prevalence of FSD in the TL women was around 50% in comparison to 27.4% in the infertile group and 8.5% in the condom group. Many other studies have also confirmed that women with infertility had high sexual dysfunction [33–35]. Omani-Samani [36] carried out a meta-analysis study to estimate the prevalence of FSD among Iranian infertile women. The findings suggested that more than 64% of the participants reported sexual dysfunction. It is well-known that the risk of FSD in infertile women is higher because the tendency of having sexual intercourse is strongly affected by pregnancy. Infertile couples define sex as a clinical tool, which should happen on certain days of the month, instead of an act of love [37]. It has, on the other hand, been suggested that, in some cases, infertility might be a result of sexual dysfunction [38]. The results of studies that have assessed the effects of TL on SF are controversial. Surprisingly, Smith et al. [12] and Li et al. [11] observed positive effects of sterilization on SF, may be due to the reduced fear of getting pregnant. In contrast, Kunkeri et al. [39] and Jahanian Sadatmahalleh et al. [9] reported higher FSD in women undergoing TL as compared with the control group.

According to the proven effects of infertility on the quality of SF [33], this finding was unexpected. But a possible explanation for this might be “post-tubal sterilization syndrome” that causes a decrease of libido, menstruation disorders, pelvis pain, dyspareunia, and depression [40].

SF is complex and involves interactions of many factors, including emotional connection, body image, and other elements such as cultural differences, ethnicity, misinterpretation of religious codes, personal belief regarding her role as a woman, and social pressures [41, 42].

Different results mentioned above may imply that the relationship between TL and sexual function is a complex process influenced by multiple factors, including cultural, ethnic and religious differences. After TL, numerous parameters can involve in increased sexual dysfunction such as change in self-concept and understanding of her existence as a woman; this carries a high meaning load in some cultures and societies.

In societies such as Iran, mothers are highly respected, and the picture a woman holds of herself is to a high degree dependent on her fertility and motherhood ability. It conveys to her feelings of satisfaction, perfection and value. Hence, despite the women’s voluntary participation in the sterilization programs, undergoing TL seems to produce in some of them an unattractive and imperfect picture, which after a while can be reflected in the appearance of sexual dysfunction and regret from this operation [9].

Furthermore, an individual’s feeling of the irreversibility of the operation may result in tempting thoughts, which are influential in sexual function. Since, a woman cannot become pregnant after TL, some couples may think that it is not really necessary to have a sexual life any more [43]. As a result, they may lose interest in sexual activity but continue to do it because of their partner's desire. The lack of knowledge of women in reaching satisfaction and orgasm in their sexual relationships are other main reasons of women’s sexual dysfunction and low QOL.

Furthermore, there is a general belief in some societies that sex is more necessary for men than for women and that women have to fulfill men’s needs [44].

Overall, it seems that having a discussion about sexual matters with others, even with the spouse and doctor is centuries-long taboo [39].

In the study of Jahanian Sadatmahalleh et al., the prevalence of FSD in the TL group was reportedly 63.4% in comparison with 40.8% in the control group [14]. Being completely informed about TL procedure and its complications, and having access to other contraceptive options prior to the operation may be helpful in avoiding future dissatisfaction.

### Quality of life status

We found that QoL (both physical and mental) in women with TL was significantly lower than in the other groups.

Several reports have shown that infertility is associated with decreased physical and mental health [45, 46]. The effects of the stage of infertility process, gender, and the relationship quality on marital relationship and QoL have been indicated; infertility causes many problems such as dissatisfaction, stress, sadness, insomnia, increased/decreased appetite, increased smoking, social pressure, exposure to questions of curiosity about having children, avoiding the places where children are, losing the privacy of sex life, having sex in some planned days just for reproduction, interruption in work life and the high cost of treatment. Therefore, all these can contribute to the low QoL scores in infertile women [47].

Numerous studies on the impact of TL on women's QoL have revealed conflicting results. For instance, Pauls et al. [13] conducted a study to summarize the recent literature on sexual function following benign gynecological surgeries, including TL. They concluded that the QoL and sexual function in the majority of the cases benefit from surgical interventions. However, Li et al. [11] did not find such associations. Some studies have demonstrated that TL has a negative impact on QoL [9, 10]. Monga et al. [48] interviewed 18 infertile couples and 12 couples seeking elective sterilization and reported that the total marital adjustment and quality of well-being scores of the women seeking infertility treatment were lower than in women with elective sterilization.

Discrepancies in QoL and sexual function conditions after TL surgery can be the result of different definitions of TL in various cultures. In societies with traditional cultures, self-satisfaction, value, and the image that a woman holds of herself are closely related to their motherhood ability. Although TL seems to be a voluntary choice at first, it might induce a feeling of guilt in some women. Therefore, sexual dysfunction, a decrease in QoL, and regret for having done this operation might happen after a while [9]. In sum, due to the growing demand for TL surgery as a permanent approach of contraception, the number of women who regret choosing this method has increased [49]. Regret is associated with sadness, pain, harm, affliction, anxiety, and displeasure, all of which affect the QoL [50].

Despite the fact that multiple psychological and physical factors like FSD can affect a woman’s QoL [51], many physicians refuse to look for details about sexual function. In addition, women often do not perceive it as a disorder and never discuss this with their doctors, so many of them may suffer in silence for years [39]. Women should view sexual function as a biological and enjoyable demand and they should be free to talk about their sexual problems, especially with their husband and doctor. This cannot be achieved except through education and counseling [39].

In spite of the well-known adverse effects of infertility on women's health, the results of this study imply the relationship between TL and the poor quality of various aspects of a woman’s life. The findings would provide a great value for fertility and childbearing in societies like Iran. The study distinguishes the importance of making the right decisions about anything that may affect fertility more than previous studies. Nonetheless, it was limited by the absence of sufficient information about the history of QoL, FSD, and the mental health of individuals before TL surgery. A larger sample size would be helpful to achieve more significant results. Another limitation was the high refusal rate of 36% for participation, which may impact the results of the research in ways not possible to determine. However, the main reason given for not participating in it was the statements concerning a lack of time (I don’t have time to complete the questionnaire or the numbers of questionnaires are high).

## Conclusion

The present study assessed sexual function, QoL, and psychological health in women undergoing TL and showed that TL women are more disrupted comparing to infertile and fertile groups. To our knowledge, it is for the first time that such a study is conducted in Iran. The findings have significant implications and suggest a need for investigating the side effects of TL more carefully, as it may not be a safe method for contraception. Overall, the study provides profound insight into the importance of a comprehensive consultation before TL.

## Data Availability

The data sets used and analyzed for the current study are available upon reasonable request of the corresponding author Dr. Shahideh Jahanian (shahideh.jahanian@modares.ac.ir).

## Abbreviations

FSFI: Female Sexual Function Index
FSD: Female Sexual Dysfunction
HADS: Hospital Anxiety and Depression Scale
QoL: Quality of life
BMI: Body Mass Index
TL: Tubal ligation
